# Comparative transcriptome analysis revealed differential gene expression in multiple signaling pathways at flowering in polyploid *Brassica rapa*

**DOI:** 10.1186/s13578-021-00528-1

**Published:** 2021-01-12

**Authors:** Janeen Braynen, Yan Yang, Jiachen Yuan, Zhengqing Xie, Gangqiang Cao, Xiaochun Wei, Gongyao Shi, Xiaowei Zhang, Fang Wei, Baoming Tian

**Affiliations:** 1 School of Life Sciences, Zhengzhou University, Zhengzhou, 450001 Henan China; 2 Henan International Joint Laboratory of Crop Gene Resources and Improvements, School of Agricultural Sciences, Zhengzhou University, Zhengzhou, 450001 Henan China; 3 Institute of Horticultural Research, Henan Academy of Agricultural Sciences, Zhengzhou, 450002 Henan China

**Keywords:** Polyploidy, Transcriptome, Flowering, Regulatory pathway, Transcriptional factors, *Brassica rapa*

## Abstract

**Background:**

Polyploidy is widespread in angiosperms and has a significant impact on plant evolution, diversity, and breeding program. However, the changes in the flower development regulatory mechanism in autotetraploid plants remains relatively limited. In this study, RNA-seq analysis was used to investigate changes in signaling pathways at flowering in autotetraploid *Brassica rapa*.

**Results:**

The study findings showed that the key genes such as *CO*, *CRY2*, and *FT* which promotes floral formation were down-regulated, whereas floral transition genes *FPF1* and *FD* were up-regulated in autotetraploid *B. rapa*. The data also demonstrated that the positive regulators *GA1* and *ELA1* in the gibberellin’s biosynthesis pathway were negatively regulated by polyploidy in *B. rapa*. Furthermore, transcriptional factors (TFs) associated with flower development were significantly differentially expressed including the up-regulated *CIB1* and *AGL18,* and the down-regulated *AGL15* genes, and by working together such genes affected the expression of the down-stream flowering regulator *FLOWERING LOCUS T* in polyploid *B. rapa.* Compared with that in diploids autotetrapoid plants consist of differential expression within the signaling transduction pathway, with 13 TIFY gens up-regulated and 17 genes related to auxin pathway down-regulated.

**Conclusion:**

Therefore, polyploidy is more likely to integrate multiple signaling pathways to influence flowering in *B. rapa* after polyploidization. In general, the present results shed new light on our global understanding of flowering regulation in polyploid plants during breeding program.

## Background

The biological and genetic advantages of polyploids relative to diploids are vast, and most studies conclude that polyploids often possess novel traits that are not present in the diploid progenitors [1, 2]. These novel traits, such as the increase in organ size and biomass, resistance to pests and changes in flowering time could allow polyploids to enter new environmental niches [3–5].

The effects of polyploidization on plant development have been extensively studied at the transcriptome level [6, 7]. Gene expression may be significantly altered as ploidy level changes, and thus contributed to changes in phenotypic traits. For example, autotetraploids in *Betula platyphylla* exhibited increased breast-height diameter, volume, leaf, fruit, and stomata size in autotetraploid plants. Further analysis of this study revealed that such increases in morphological traits might have been contributed by the significant up-regulation of indole acetic acid (IAA) and ethylene signaling transduction hormones [8]. Additionally, in apple plants (*Malus* d*omestica*) a dwarf phenotype was observed for synthetic autotetraploid plants. It was indicated that hormones including IAA and brassinosteroid (BR) were also significantly decreased; which indicated a partial interruption of the IAA and BR signal transduction pathways in the polyploidy apple plants [9]. Furthermore, studies have indicated genomic instabilities that occurred during meiosis in some plants might also play some roles in genetic diversities [10]. These genome instabilities occurring during meiosis usually affect neopolyploids [11]. The process by which neopolyploids survived with low diversification and reproductive disadvantages including flowering is still poorly studied [7].

Over recent years, various scientific progress has been made to decipher the molecular basis of vegetative to reproductive transition in polyploid plants [12]. Most alternation during flowering are related to epigenetic and genetic mechanisms. Flowering might be regulated by a complex network of genetic pathways, which were strongly responsive to endogenous changes and environmental stimuli [13]. In allotetraploid *Brassica* and *Arabidopsis* plants, plants with delayed flowering from a late and early flowering parent were mostly observed [14]. In addition, the flowering and bolting times for autotetraploid were delayed by 8.3 and 11.2 days compared with the wild type plants [15]. Furthermore, the bolting time and rosette growth in *Arabidopsis* ploidy plants were remarkably delayed at 22, 23 and 26 days in tetraploid, hexaploid and octoploid, respectively [16]. These data implied that changes in flowering variation might be related to subtle alternation in specific signaling or genetic pathways after the induction of polyploidization. Moreover, flowering date and height in alfalfa autotetraploid were associated with CONTANS-like genes [17]. However, with the numerous studies concerning flower development in autotetraploid plants, to best of the authors knowledge, little information is available about the cascade of regulatory pathways and interaction of genes related to the delay in flowering after polyploidization.

In the present study, transcriptome analysis was used to investigate the genome-wide changes of gene expression regulation on flowering in autotetraploid *B. rapa* in comparison with its diploid counterparts, which would further deepen our understanding of polyploidy-associated effects on flowering regulation.

## Results

### Phenotypic characteristics in autotetraploid *B. rapa*

**Fig. 1:**
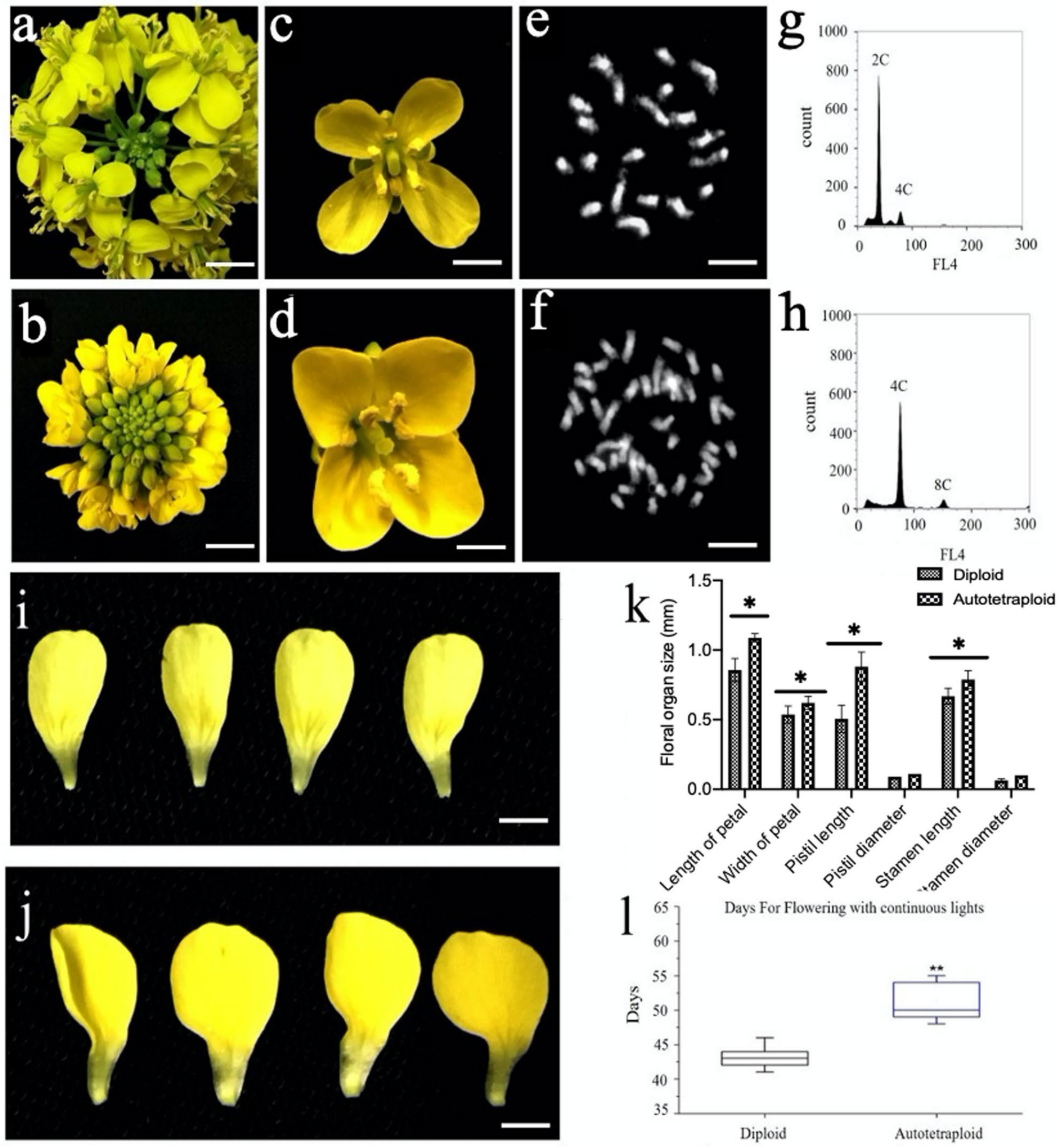
Phenotypic analysis, chromosome number and flow cytometric analysis of the colchicine-induced *B. rapa* plants. **a**, **b** flower of diploid *B. rapa* and the enlarged flower of *B. rapa* plant. Bar = 1 cm. **c**, **d** Open flower of the diploid and autotetraploid *B. rapa*. **e**, **f** Exactly 20 chromosomes in diploid *B. rapa* and 40 chromosomes counted in autotetraploid *B. rapa*. Bar = 10 μm. **g**, **h** Peak distribution of nuclear DNA content in autotetraploid in comparison with diploid *B. rapa.* **i**, **j** differences in petal sizes between diploid and autotetraploid *B. rapa*. **k** Measurements of floral tissues between diploid and autotetraploid. **l** Depiction of floral transition between the diploid and autotetraploid. Asterisks indicate the following criteria of significances: *p ≤ 0.05

As shown in Fig. 1, the induced polyploid *B. rapa* plants consisted of significantly enlarged flowers (Fig. 1b, d), when compared to its diploid progenitors (Fig. 1a, c). The chromosome counts for autotetraploid *B. rapa* plants displayed 40 chromosomes after genome duplication indicating a level of tetraploidy (Fig. 1f) whereas diploids plants consist of a somatic chromosome set of 20 (Fig. 1e). In addition, flow cytometric results indicated a two-fold increase in DNA content in autotetraploid (Fig. 1h), compared with that of DNA content observed for the diploids (Fig. 1g). Moreover, floral transition in the induce plants was delayed by 7–10 days in comparison with the diploids (Fig. 1l). The results obtained here demonstrate the induced tetraploid *B. rapa* had enlarged flower organs and postponed flowering after polyploidization (Fig. 1k, i).

### Analysis of differentially expressed genes related to flowering

**Fig. 2:**
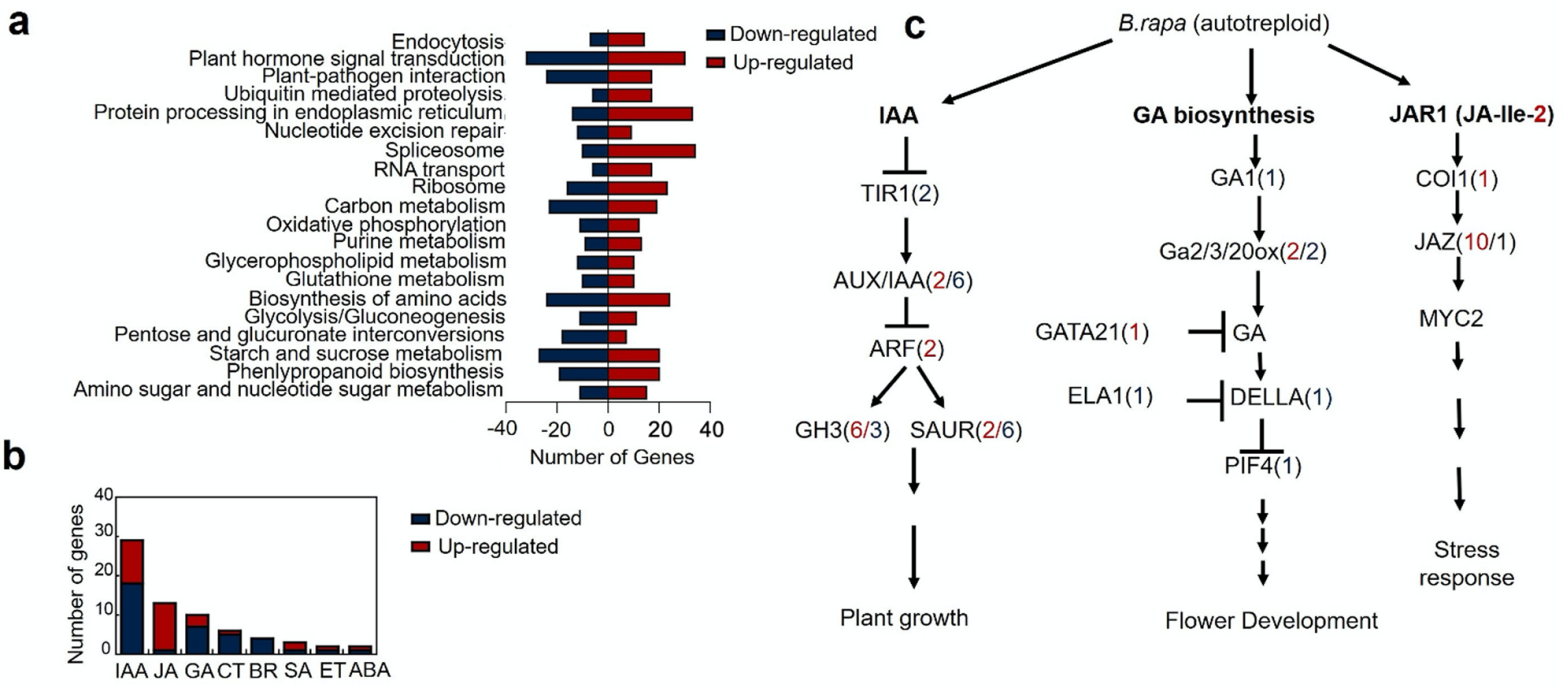
Plant hormone signal transduction pathway altered after polyploidization. **a** Number of DEGs enriched in KEGG pathways. **b** The number of hormones signal-related DEGs altered in polyploidy *B. rapa*—*BR* brassinosteroid, *GA* gibberellin, *ABA* abscisic acid, *ET* ethylene, *IAA* auxin, *JA* jasmonate, *CTK* cytokinin, *SA* salicylic acid. **c** Simplified model representing IAA, and GA, JA signal transduction induced by polyploidy *B. rapa*. The numbers in parentheses correspond to the number of up-regulated (red) and down-regulated genes (blue)

As both diploid and tetraploid *B. rapa* have the same number of chromosome set, we predicted that the changes in flower development of the tetraploid *B. rapa* may be associated with altered regulatory pathways. To examine genes expression profiles involved in flower development after polyploidization*,* cDNA libraries were constructed. RNA-seq analysis revealed that 4601 genes consisting of a probability cut of ≤ 0.05 to be significantly differentially expressed. Functional analysis was performed to identify KEGG pathways affected after polyploidization. KEGG pathway analysis was firstly performed for all DEGs. The results showed approximately 35% DEGs were assigned to 50 biological pathways with plant hormone signaling transduction pathway (69) enriched with the largest number of DEGs. Biosynthesis of amino acids (48), and protein processing endoplasmic reticulum pathways (47) were also overrepresented with DEGs (Fig. 2a). In addition, flower time-related pathways were also assets to investigate the delay in flowering observed in the autotetraploid plants. Interestingly, all flower-time pathways consisted of differentially expressed genes except the vernalization pathway which was relatively stable. These results indicated that multiple pathways associated with flower development were substantially altered in autotetraploid *B. rapa*.

### Hormone signaling pathways differentially regulated in polyploid *B. rapa*

KEGG enrichment analysis showed that 69 DEGs involved in plant hormone signaling transduction pathways were altered in the polyploidy plants (Fig. 2b, Additional file 1: Table S1). More specifically, 42% (29) were associated with auxin signaling, 18.8% (13) with jasmonate (JA), 14.5% (10) with gibberellin signaling (GA), 8.7% (6) with cytokinin (CTK), 5.8% (4) with Brassinosteroid (BR), 4.3% (3) with salicylic acid (SA), 2.9% (2) with abscisic acid (ABA) and 2.9% (2) with ethylene (ETHY) signaling (Fig. 2b). Genes enriched in the auxin pathway included 9 *GH3* genes, 2 auxin response factors (*ARF*) genes, 2 *GRR-1/TIR1* genes, 8 genes that were downstream response to *AUX/IAA* and 8 *SAUR* genes were differentially regulated for this pathway (Fig. 2c). From the 29 genes regulated in this pathway, 12 genes were up-regulated and 17 down-regulated. In the JA signaling pathway, two up-regulated genes encoding JAR1 protein, 10 up-regulated genes encoding TIFY proteins and one gene up-regulated which encoding *Coronatine-insensitive protein 1 COI1* were significantly altered after polyploidization. Enrichment analysis revealed that GA signaling genes DELLA (*RGL2*) and *PIF4*, were both down-regulated. In addition, RNA-seq data showed that 8 genes involved in GA biosynthesis were differentially expressed in polyploid *B. rapa*, which included *GA1*, *GA2ox7*, *GA20ox5*, *GA3ox4*, *GA2ox8*, *CARBON METABOLISM INVOLVED* (*GNC*), *GNC-like* (*GNLI*), *CYTOCHROME P450 714A1 (ELA1*). Elevated expression of *GA20ox5*, *GNCLI,* and *GA3ox4* was up-regulated in polyploid *B. rapa,* whereas genes *GA2ox7, GA1, GA2ox4, ELA1* were down-regulated.

Further, after polyploidization, the CTK pathway consisted of five down-regulated genes in the *Arabidopsis response regulators* Family such as *ARR2, ARR4, ARR6,* and two *ARR5* and one up-regulated gene (*ARR8*). Meanwhile, BR signaling had four down-regulated genes including *SERK2*, *BIN2*, *BIL*1 and *Protein kinase-related gene*. SA signaling consists of two up-regulated *NPR* genes including *NPR* and *NPR6/BOP2*. In addition, *PRP1* was down-regulated in this pathway. For the process of ABA signaling pathway, *AB15* and *SnRK2* were down- and up-regulated, respectively. While three PP2C genes were all up-regulated. Furthermore, for ETHY signaling *EIN3*, *CTRI* and *ERS2* were down- and up-regulated, respectively. These results indicated that polyploidization (chromosome doubling) could lead to a distinct hormone signaling regulation, which may play an important role in the flower development or floral organ growth process in autotetraploid *B. rapa*.

### Genes related to floral organ development

To further investigate the change in the flower enlargement of autotetraploid plants, all genes related to organ size were assessed. Genes such as *GROWTH-REGULATING FACTORS* (GRFs) and *GRF-INTERACTING FACTORs* (GIFs), A*UXIN-REGULATED GENE INVOLVED IN ORGAN SIZE* (*ARGOS*) and AP2/ERF type transcription factors *AINTEGUMENTA* (*ANT*) were not differentially expressed. However, one known gene in *Arabidopsis* which promote floral organ growth KLU/CYP78A5 was differentially expressed. After polyploidization, two KLU/CYP78A5 genes were up-regulated (Additional file 1: Table S2). These results indicate that the change in organ size may not have occurred due to late floral phenotype observed. However, the floral enlargement occurred may occur due to polyploidization.

### Identified unigenes related to flower-time regulation in *B. rapa*

**Fig. 3:**
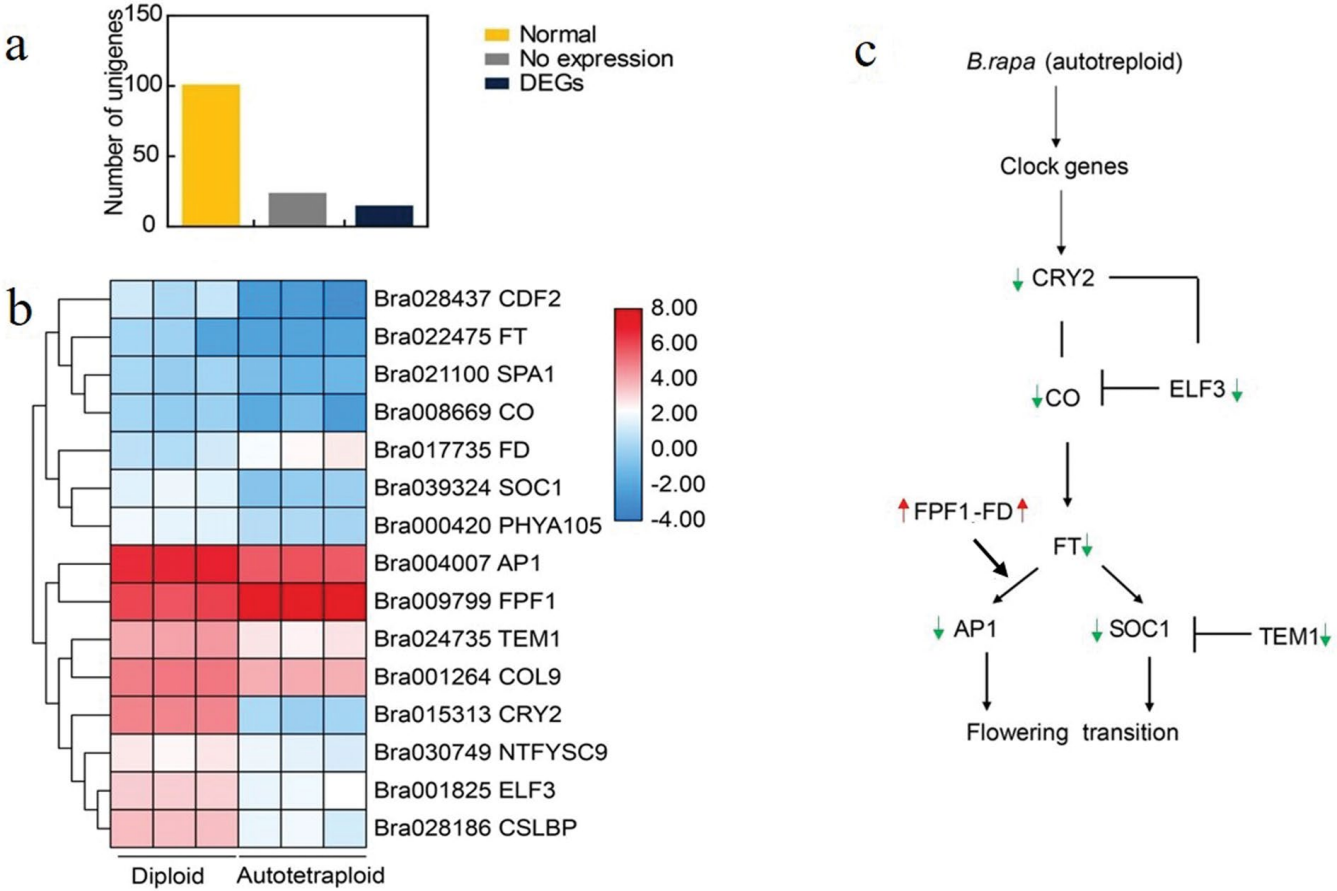
Expression patterns of altered genes in the photoperiod/circadian clock pathway. **a** The number of photoperiod/circadian clock genes represented in *B. rapa* genome. **b** The expression level of DEGs concentrated in the photoperiod/circadian clock pathway. **c** Pathway analysis of genes that interact, activate and repress the function of *FT*. The expression patterns are normalization log_2_ transformation FPKM values. Green arrows represent down-regulation genes, whereas red arrows indicate up-regulation genes

In addition to changes in autotetraploid *B. rapa* floral growth, the phenotypic analysis indicated a delay in floral transitioning. Thus, to study the probable cause for the delay flowering observed in autotetraploid *B. rapa*, flowering related pathways were analyzed including photoperiod/circadian clock pathway, meristem response and development, and vernalization and autonomous pathways. Using homologs from *A. thaliana,* as a query, 348 genes including 70 transcriptional factors were identified to be involved in flowering specific pathways in *B. rapa* (Additional file 1: Table S3, Additional file 2: Figure S1). Interestingly, among the 348 genes, 52 genes were differentially expressed. Our data indicated that 34.6% (18 genes) of the 52 DEGs related to flowering were concentrated in the photoperiod/circadian clock pathway (Fig. 3a, Additional file 1: Table S4). Among these, 12 flowering-related genes were significantly down-regulated including *CYCLING DOF FACTOR 2* (*CDF2), NUCLEAR TRANSCRIPTIONAL FACTOR Y SUBUNIT C-9* (*NFYC*3)*, SUPPRESSOR OF OVEREXPRESSION OF CO1* (*SOC1)* a transcriptional activator of flowering control, *APETALA 1A* (*AP1*)*, EARLY FLOWERING 3* (*ELF*3)*, TEMPRANILLO 1* (*TEM1*)*, CRYPTOCHROME 2* (*CRY*2)*, GLYCINE-RICH RNA -BINDING* (*CCR1*)*, ZINC FINGER PROTEIN CONSTANS LIKE 9* (*COL 9*)*, CHLOROPLAST STEM-LOOP BINDING PROTEIN* (*CRB*)*, SUPPRESSOR OF PHYA 105* (*SPA*1)*, SPA1 RELATED 3* (*SPA3*), and *LHY/CCA1-like 1* genes. In addition, *FLOWER PROMOTING FACTOR 1 (FPF1)*, *FLOWERING LOCUS D (FD)*, *EARLY PHTOCHROME RESPONSIVE1* (*EPR1*) *and TWIN SISTER FT* (*TSF*) were significantly up-regulated in autotetraploid *B. rapa* (Fig. 3b). Furthermore, the initiator of flowering *FLOWER LOCUS FT (FT, Bra022475*) was also down-regulated (Fig. 3c). Thus, such results imply that the altered expression of photoperiodic/ circadian genes was distinct after polyploidization, indicating a probable cause of delay floral transiting.

For the vernalization and autonomous pathway, only four genes were differentially expressed including *FRIGIDA like* (*FRL*) and *RNA -BINDING protein (AtCRP7*) was found down-regulation in *B. rapa*. Many genes involved in the vernalization pathway, *EMBRYONIC FLOWER 2* (*EMF2*), *VERNALIZATION 2* (*VRN*2)*,* and *FRIGIDA* (*FRI)*, were identified to be normally expressed between the autotetraploid and diploid *B. rapa.* However, *FLOWERING LOCUS C* (FLC) an important player of the vernalization pathway consists of four genes in *B. rapa*, but only *FLC5* was differentially expressed, up-regulated. These results imply that the vernalization and autonomous pathways were not affected by the change in ploidy level. However, the up-regulation of *FLC5* a known floral repressor in *B. rapa* may have contributed to the delay in flowering.

### Expression of transcription factors affected in polyploid *B. rapa*

**Fig. 4:**
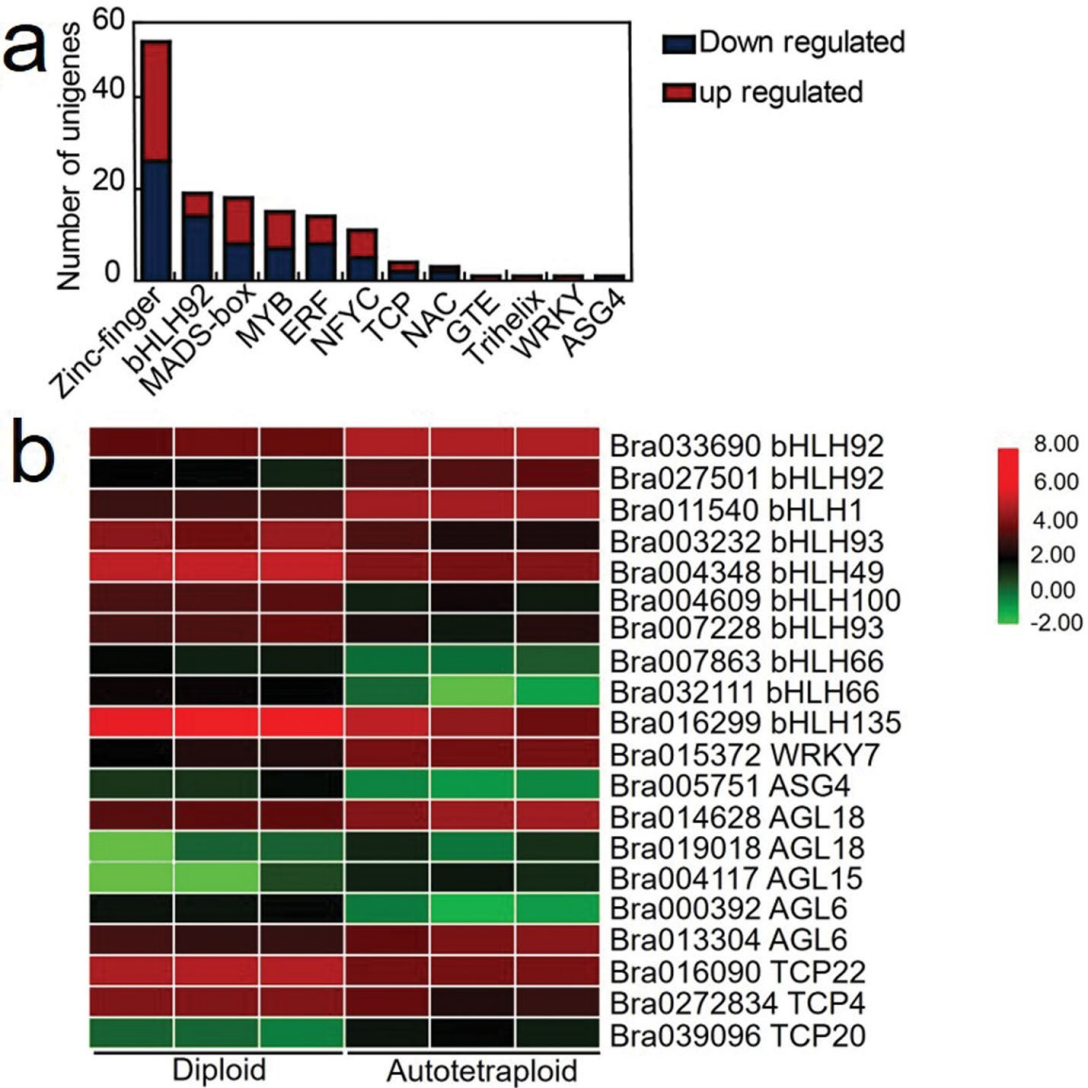
Differential expressed transcriptional factors (TF) in autotetraploid *B. rapa*. **a** Number of transcriptional factors related to flower-time transitioning. **b** Heatmap representing TFs related to flowering time

**Fig. 5:**
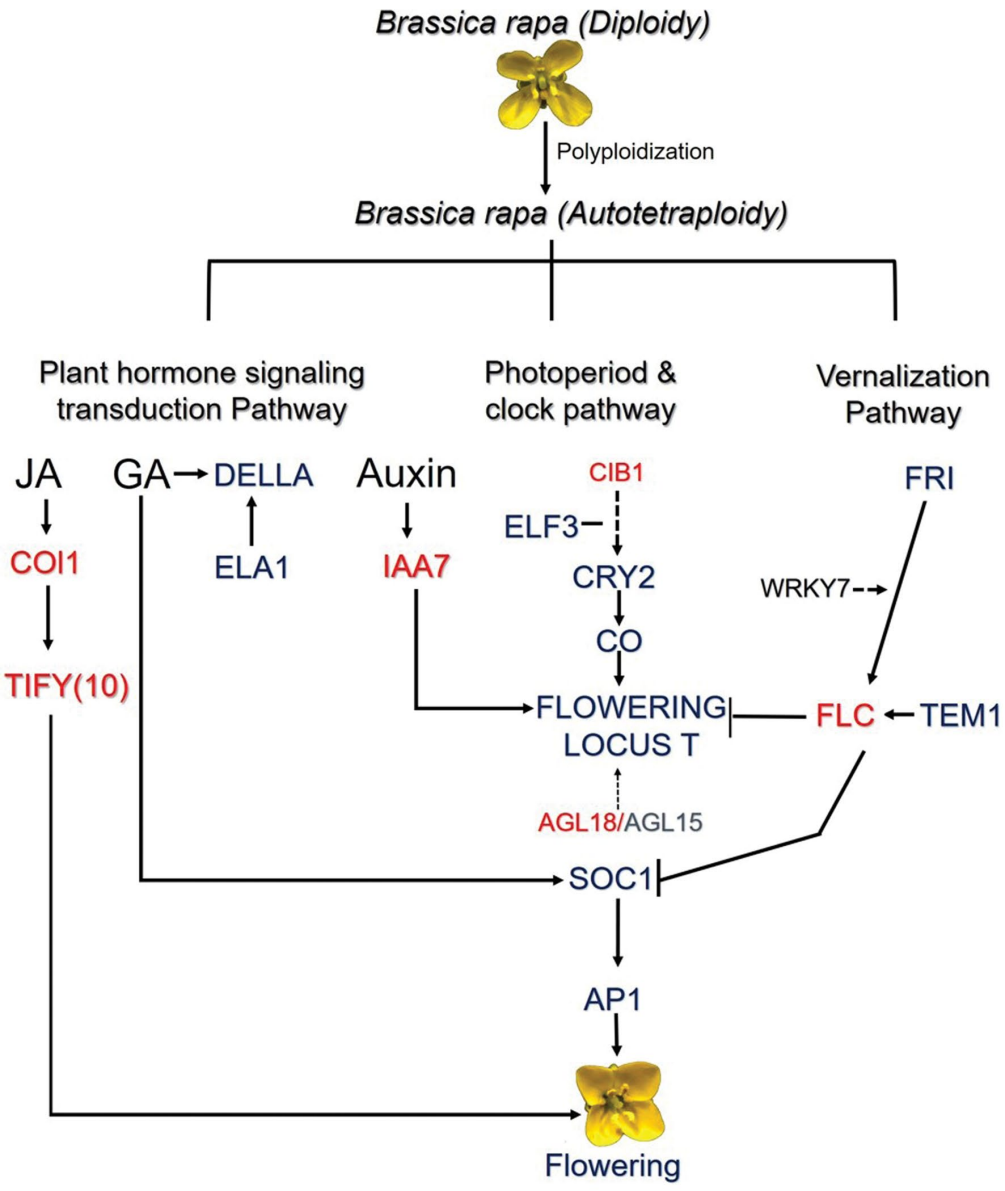
Proposed working model of the flowering cascade observed in autotetraploid *B. rapa*. Blue representing down-regulated genes while red represents up-regulated genes

To further understand the delay in flowering of autotetraploid *B. rapa,* we identified the transcriptional factors (TFs) that were associated with flower development and floral transition. Over 135 TFs were differentially expressed with 21 TFs associated with flowering; including 12 down-regulated and 9 up-regulated genes between autotetraploid and diploids *B. rapa* (Fig. 4a, Additional file 1: Table S5). For flowering, transcriptional factors bHLH families were the most represented group of differentially expressed genes consisting of ten genes (Fig. 4b, Additional file 1: Table S5). For bHLH, 3 TFs were up-regulated, two *bHLH 92* and *CIB1/bHLH1*.Whereas, 7 TFs displayed down-regulation including two *bHLH93,* two *bHLH66, bHLH49, bHLH100* and *bHLH135.* In addition, MADS-Box TFs two *AGL18 and AGL17* were down-regulated, whereas *AGL15, AGL19*, *AGL6* were up-regulated*.* Transcription Factors TCP class I/II consisted of 4 genes, two up-regulated genes *TCP2* and two down-regulated genes *TCP22* and *TCP4*. Furthermore, WRKY TFs only had one gene which was affected after polyploidization, including *WRKY7* (up-regulated) (Fig. 4b). Taken together, these results indicated that transcriptional factors associated with flowering were also affected after polyploidization in *B. rapa*. In general, a working model was proposed to reveal the changes in regulation pathways at flowering in autotetraploid *B. rapa* (Fig. 5).

### Verification of RNA-seq by qRT-PCR analysis

**Fig. 6:**
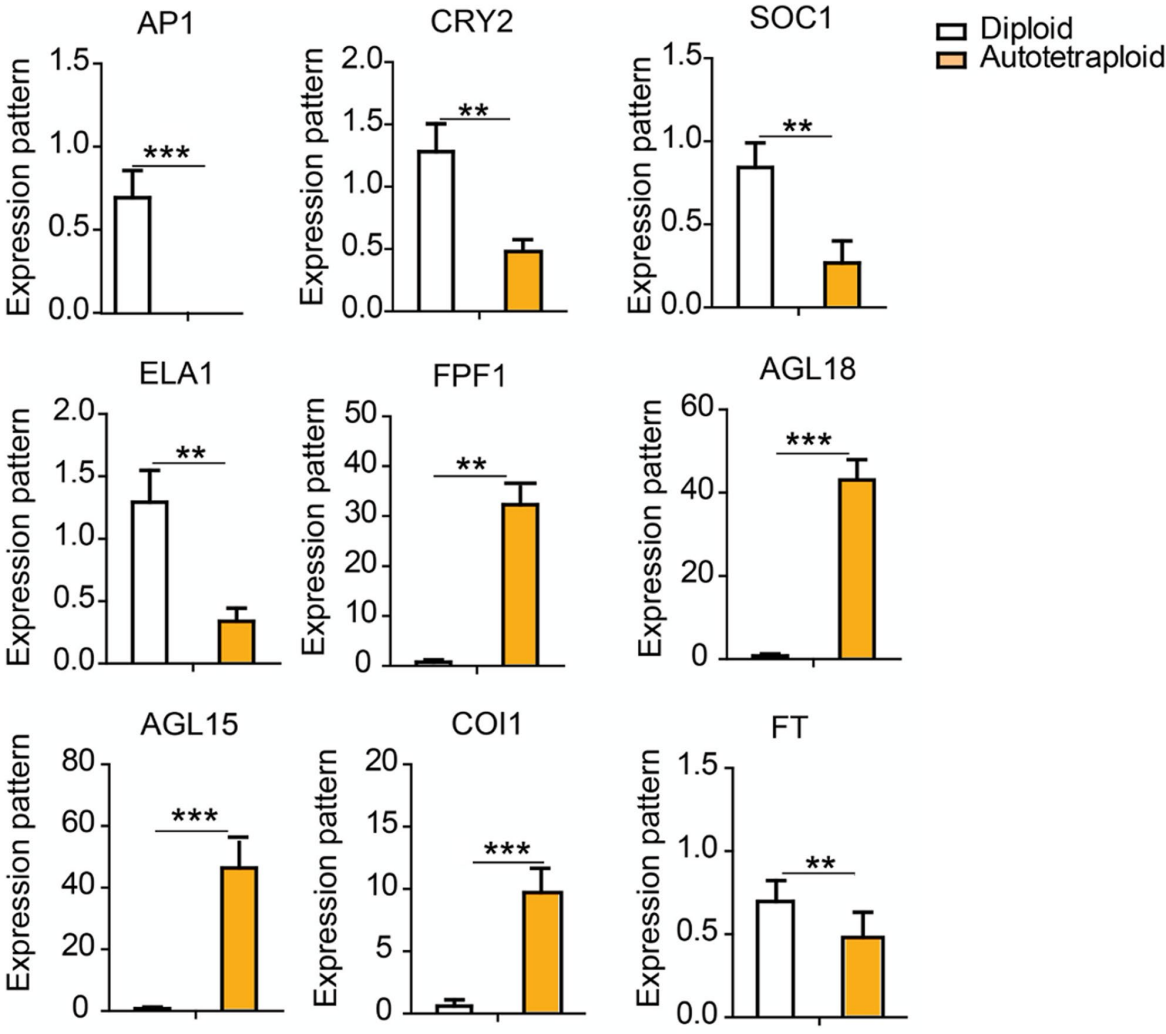
Confirmation of RNA-seq data using qRT-PCR analysis. For relative expression, three independent biological replicates of each sample were performed. Asterisks indicate the following criteria of significances: **p ≤ 0.05 and ***p ≤ 0.001

To verify the RNA-seq data, nine DEGs were selected for qRT-PCR analysis. The results showed that seven out the nine DEGs displayed similar expressions to the RNA-seq data in both diploid and autotetraploid (Fig. 6; Additional file 2: Figure S2). *AGL15* showed dissimilar expression patterns with the RNA-seq data. *AGL15* was highly up-regulated after qRT-PCR analysis, whereas the expression was decreased for the RNA-seq results. Despite the dissimilar expression displayed for *AGL15*, these results indicated that the RNA-seq data were reliable and the results were highly reproducible.

## Discussion

Numerous studies on the flower formation of *A. thaliana* and other model species uncovered several critical regulators for flower development. The polyploid induction results in enlargement of floral organs and in most studies delay flowering for majority of polyploid plants [16]. However, to date most researches have not indicated specific signaling pathways which might affect the observed flowering delay and enlargement in polyploid plants. In this study, the polyploidy-induced changes at flowering in tetraploid *B. rapa* and changes in signaling transduction pathways were investigated using RNA-seq technologies.

### DEGs changes in multiple signal transduction pathway affect floral transitioning in autotetraploid *B. rapa*

Numerous plant hormones have been shown to participate in transition of flowering including JA*,* GA, auxin*,* ABA and cytokinin [18, 19]. For floral transition, the role of GA pathway has been thoroughly investigated [20–22]. GA exerts its biological functions on floral transition by degrading DELLA proteins and activating the key floral transition integrators (*SOC1, AGL24,* and *LFY*) [16]. In *A. thaliana*, mutation in *GA1* locus displayed a severe delay in flowering [23]. After polyploidization in *B. rapa*, the *GA1*, DELLA (*RGL2*) and *PIF3* genes were severely down-regulated in autotetraploid *B. rapa*. Moreover, the up-regulation of ABA associated genes *PP2C*s block the generation of some *SnRK2* genes causing different growth periods between two *B. rapa* accessions (Jin Wawa and Xiao Baojian), during continuous vernalization periods [24]. However, in the two plants used in this study, autotetraploid *B. rapa* exhibited an up-regulation for *SnRK2* after polyploidization compared to diploid *B. rapa*. Additionally, the PP2C genes were up-regulated in this study. Plants hormones are known to interact with flowering time genes to effect flowering in majority of plants. JA receptor gene *COI1* negatively regulates flowering through repressing the expression of *FT* [25]. In polyploid *B. rapa COI1* gene was up-regulated, it is probable that the up-regulation of *COI1* may repress the expression of *FT.* ABA and *DELLAs* both interact with *FLCs* to cause alternation during flowering. Therefore, JA, ABA, and GA signaling may participate in regulating the floral transitioning in autotetraploid *B. rapa.*

In most flowering plants, the timing of flowering is primarily influenced by numerous flowering time genes. Cryptochrome 2 one of many positive integrators of flowering time, interacts with *CO* protein to enhance its stability. While *CO/FT* expression is a core link between the photoperiod induce pathway [26]. In *B. rapa*, plants under high ambient temperatures consist of reduce expression of *FT* and delay flowering via a mechanism associated with Histone variant (*H2A.Z*). The data here indicated that the expression of *FT* was low but not significant, while *CRY2* was significantly down-regulated with *CO* and *SOC1* displaying stable and down-regulated expressions, respectively. Both *FT* and *SOC1* are known to strongly initiate flowering, thus the differential regulation of such genes could alter flowering in plants. Furthermore, the regulation *FT* interacts with *AP1*. The expression of *AP1* after polyploidization was down-regulated. The common *AP1* down-regulation in floral meristems is commonly associated with late flowering [27]. Moreover, *FLC* a central player in the vernalization pathway consists of four copies in the *B. rapa* genome [28]. Unlike, *FLC1*, *FLC3,* and *FLC2* which are known to delay flowering in *B. rapa*, *FLC5* is a relatively weak regulator in *B. rapa*. For this study, *FLC5* was slightly up-regulated but not significant. The expression of *FLC* is positively regulated by *FRI* an causative genes in the vernalization pathway [29]. Between the diploid and polyploid plants the *FRIGIDA* gene were not differential expressed but *FRIGIDA*-like genes in polyploid *B. rapa* were differentially expressed. Flowering time in *B. rapa* is an complex mechanism due to duplicated genes. Our results suggest the relationship between flowering time pathways and plant hormones signaling trandcution pathways may contribute to the delay in flowering observed. However, genetic interaction between specific pathways is complex to understand in autotetraploid *B. rapa*.

### Effects of TFs on flowering time variation in autotetraploid *B. rapa*

MADS-domain transcription factors may positively and negatively regulate the vegetative to reproduction transition in plants. When *AGL15* and *AGL18* were over-expressed, it significantly reduced the expression of *FT* and participated in the late development of flowers. *AGL15* and *AGL18* were down-and up-regulated, respectively. The results presented here were inconsistent with the previous research which indicated that the expression of *AGL15* in most cases consisted of similar expression with *AGL18* [30]. These results suggest that after polyploidization, both *AGL18* and *AGL15* displayed independent expression patterns despite the overlapping functions shared between the MADS-box genes [31]. The high expression of *AGL18* may have interact with the expression of *FT* independent of *AGL15* expression*.* Additionally, one previous study indicated the overexpression of *CIB1* caused early flowering in *A. thaliana* plants [32]. *CIB1* a transcriptional factor *bHLH1* interacts with *CRY2* to regulate and activate the expression of *FT* but independent of each other. In this study, the expression of *CRY2* was significantly down-regulated and *CIB1* was up-regulated. Most transcriptional factors outline in this study had an essential role in the regulation of polyploid *B. rapa* flowering variation*.*

### The probable cause of floral growth in autotetraploid *B. rapa*

Flower size involves the timing of cell proliferation arrest within developing floral organ primordia [33]. The control of such mechanism can involve plant hormones [33–38] and genes related to organ growth [39]. Pathways such as Auxin, Brassinosteriod, cytokinins and ethylene, when regulated contribute to numerous functions including, cell proliferation and organ growth. However, in this study, majority of genes related to floral size such as *AUXIN-REGULATED GENE INVOLVED IN ORGAN SIZE* (*ARGOS*) or the gene encoding the AP2/ERF type transcription factor *AINTEGUMENTA* (*ANT*) and cytokinin oxidase/dehydrogenase *CKX3* and *CKX5* consist of normal expressions. The only noticeable gene TIFY/JAZ consists of an elevated expression in autotetraploid *B. rapa*. In previous studies overexpression of specific *JAZ* genes promotes an increase in grain size and increase floret numbers in plants placed under stressful conditions [40]. Additionally, our study indicated that after polyploidization, two KLU/CYP78A5 genes were elevated. An earlier study in *Arabidopsis* indicated that mutant and overexpressed lines consist of smaller and larger floral organs, respectively [41, 42]. The result indicates that, upon polyploidization, the KLU/CYP78A5 genes may affect floral size, as well as lack genetic interaction from known floral size contributors, including auxin and Brassinosteriod.

## Conclusion

In conclusion, to date studies on the mechanism related to flowering and floral organ in autotetraploid plants focus only on the comparison between the parental lines and polyploids at the phenotypic level. Polyploid are advantageous and considered more favorable than their diploid counterparts; thus, its necessary to clarity its effects on flower development. In this study, autotetraploid and diploid plants were investigated. For comparison purposes it was indicated that autotetraploid and diploids consist of various changes in flowering and floral size, which is similar to most polyploid plants. To decipher the changes in gene expression comparative transcriptome analysis was investigated. According to the results, polyploid *B. rapa* accessions exhibited significant differential regulation in multiple signaling transduction pathways. In comparing the two samples sets, keys genes within the plant hormone signal transduction pathway and flowering time pathway were notably different and the differences indicated a down-regulation for most genes and up-regulation of some for the polyploid plants. For transcription factors, *TCP4*, *bHLH* and, *WRKY* genes exhibited different expression patterns, but these different require further experimental analysis to be confirmed. Notably, KLU/CYP7845, which play a role in floral organ size, exhibited elevated expression patterns after polyploidization in *B. rapa*, which may be a new avenue for research on polyploid *B. rapa* in the future. The comparative analysis of polyploid *B. rapa* at flowering was investigated and summarized in this research, which shed light and provides a foundation for future investigation at the molecular level on the mechanisms contributing to changes in flowering of autotetraploid plants.

## Methods

### Plant materials

Autotetraploid *B. rapa* plants were generated artificially by colchicine treatment (0.1% w/v) as previously described [10]. The somatic ploidy level for all polyploid plants used in this experiment was confirmed using flow cytometry [43]. All *B. rapa* plants used in this experiment were grown in a preferred greenhouse condition of 16 h light/8 h dark cycle at temperatures of 22 °C daytime/18 °C nights. At the flowering stage, tissue samples were harvested and immediately stored in liquid nitrogen in three biological replicates for further analysis.

### Ploidy and morphological analyses of autotetraploid *B. rapa*

Floral buds in size from 0.8 to 1 mm were collected and fixed with Carnoy’s solution (ethanol: acetic acid = 3:1 v/v) for 24 h at room temperature. The samples were then stored in 70% ethanol at 4 °C until use. Anthers were removed from the floret using forceps and a dissecting needle under a stereo-microscope and incubated as previously outlined [44]. The chromosome spreads were prepared as previously described [45]. Chromosome numbers for each sample were visualized using an Olympus BX53 epifluorescence microscope equipped with a cooled CCD DP73 digital camera (Japan Olympus-life science). The floral tissues for phenotypic analysis were harvested upon opening to examined variations among plants with different ploidy levels. To assess bolting and flowering time, we measured (100 plants for each diploid and autotetraploid) the time of induction of bolting until the production of siliques.

### Total RNA extraction and mRNA sequencing

Floral buds with indefinite inflorescence were harvested from each plant in three biological replicates and stored in liquid nitrogen. Sample sets for each genotype were finely ground in liquid nitrogen and total RNA extracted using a modified TriZol (Invitrogen) reagent method with on-column DNase treatment. The poly A + RNA samples collected were annealed, reverse transcribed and ligated to adapters which were complementary to sequencing primers. Further library construction was conducted by the Biomaker Institute (Beijing, China), which included the following procedures PCR amplification and construction of libraries for RNA-seq. The RNA libraries were sequenced on Illumina HiSeq 2000 (Illumina, San Diego, California USA). For subsequent analysis, the raw data were processed by base calling and stored in FASTQ files. The generated FASTQ files were deposited in the NCBI Sequence Read Archives (SRA) (https://www.ncbi.nlm.nih.gov/sra) with the accession number SRP104015.

### Data analysis: quality control and mapping of raw reads

For data analysis, the FASTQ sequences files were filtered and trimmed similar to procedures as previously outlined [10]. The clean reads were aligned to the reference’s genome of *B. rapa* (BRAD http://brassicadb.org/ v.1.5) using the sequence alignment software TopHat 2 (v2.1.1) [46, 47]. The alignment parameters required that each read aligned to the reference’s genome sequences at least 75–80% of the inserted base pairs. Reads with multiple mismatches were excluded from the annotation process, only reads with perfect matches and one mismatch was further analyzed and annotated based on the reference’s genome. Transcript level expression for the transcriptome of *B. rapa* was evaluated using the Cufflinks suite (v2.1.0). A normalized expression level for each *B. rapa* gene model was calculated using the fragments per kilobase of Exon per million fragments mapped (FPKM) normalization method.

### Analysis of differentially expressed genes in autotetraploid vs diploid *B. rapa*

To identify differentially expressed genes (DEGs) between the diploid and autotetraploid *B. rapa* DeSeq package from the R statistical computing environment was used. The resulting probability values were adjusted using the Benjamini and Hochberg method to control the false discovery rate [48]. Stringent values of false discovery rate (FDR) ≤ 0.01 and fold change (log2 FC) ≥ 2 (p.value 0.05) as a threshold were used to identify significant differences in gene expression between the different ploidy.

### Functional classification of DEGs

For functional annotation of DEGs, bioinformatics tools were employed which included; BLAST similarity searches for gene models search against NCBI non-redundant protein (Nr) database, uniProtKB\Swissprot database, and Kyoto Encyclopedia Genes and Genomes (KEGG) (http://www.genome.jp/kegg/) was used to assign KO numbers and enrichment pathways to all map transcripts.

### Identification of orthologous flowering-related genes in *B. rapa*

The molecular and functional analysis of flowering has been extensively studied in *A. thaliana.* Since *A. thaliana* was considered as a model species for plant systems and the majority of its genes have been functionally annotated, we examined the orthologous flowering-related gene pairs between *A. thaliana* and *B. rapa*. Further, for the identified flowering-related genes in *B. rapa*, the BRAD database and chromosome data (v1.5) of Chinese cabbage were employed (http://brassicadb.org/brad/searchSynteny.php). The flowering-related genes were identified among the ten chromosomes of *B. rapa*. Furthermore, MCScanX software was used to identify and analyze the flowering-related genes between *B. rapa* and *A. thaliana*. Circos software was employed to generate the circos figure and Map Chart 2.2 was employed for mapping purposes [49]. Flowering time genes within the photoperiod/circadian clock pathway, meristem response and development, and vernalization and autonomous pathways were examined.

### Verification of RNA-seq data

RNA was extracted from the floral buds of autotetraploid and diploid plants using Trizol reagent (Invitrogen). The qRT-PCR analysis was performed using the Superscript III Platinum SYBR gene qRT-PCR kit (Invitrogen) and QIAGEN ROTOR gene 6000 (QIAGEN) system. Relative expression of interested DEGs was normalized by *β-actin* and analyzed using the Delta 2^−ΔΔCT^method. For this analysis, three biological replicates for each sample were run. Gene-specific primers were designed by Primer Premier 5.0 for 9 randomly selected genes. The amplification fragments were between 100 and 200 bp. The specificity of all primer pairs was checked with the NCBI primer design tool (NCBI) (Additional file 1: Table S6).

## Supplementary Information

**Additional file 1: Table S1.** Differentially expressed phytohormones. **Table S2.** Gene related to organ growth. **Table S3.** Chromosomal loaction of the Flower time genes among the Subgenomes of *B. rapa*. **Table S4.** Differentially expressed genes among the floral transitioning pathways. **Table S5.** Differentially expressed TF. **Table S6.** Primers pairs used for qRT-PCR.

**Additional file 2: Figure S1.** Gene duplication and collinearity analysis among Flower time genes from including *B.rapa and A.thaliana*. Lines connecting genes depict ortholog pairs diverged from the same ancestor. A1-A10 indicated *B.rapa* chromosomes, and Ath01-Ath05 displayed *A.thaliana* chromosomes. **Figure S2.** RNA-seq results of key genes in the flowering time pathway.

## Data Availability

Data availability in NCBI accession: SRP104015.

## Abbreviations

DEGs: Differentially expressed genes
GA: Gibberellin
CTK: Cytokinin
BR: Brassinosteroid
SA: Salicylic acid
ABA: Abscisic acid
ETHY: Ethylene
TFs: Transcriptional factors
