# Supplementary material for: Comparative transcriptome analysis revealed differential gene expression in multiple signaling pathways at flowering in polyploid Brassica rapa
Source: Cell Biosci. 2021 Jan 12;11:17. doi: 10.1186/s13578-021-00528-1 (PMC7802129; doi:10.1186/s13578-021-00528-1)
Supplement: Supplementary file 2 — Additional file 2: Figure S1. Gene duplication and collinearity analysis among Flower time genes from including B.rapa and A.thaliana. Lines connecting genes depict ortholog pairs diverged from the same ancestor. A1-A10 indicated B.rapa chromosomes, and Ath01-Ath05 displayed A.thaliana chromosomes. Figure S2. RNA-seq results of key genes in the flowering time pathway. [file 13578_2021_528_MOESM2_ESM.docx]

**Comparative Transcriptome Analysis Revealed Differential Gene Expression in Multiple Signaling Pathways at Flowering in polyploid *Brassica rapa***

Janeen Braynen^1, 2^, Yan Yang^1^, Jiachen Yuan^1^, Zhengqing Xie^2^, Gangqiang Cao^2^, Xiaochun Wei^3,^ Gongyao Shi^1,2^, Xiaowei Zhang^3^, FangWei^1,2*^, Baoming Tian^2*^

1. School of Life Sciences, Zhengzhou University, Zhengzhou, Henan 450001, China

2. Henan International Joint Laboratory of Crop Gene Resources and Improvements, School of Agricultural Sciences, Zhengzhou University, Zhengzhou, Henan 450001, China.

3. Institute of Horticultural Research, Henan Academy of Agricultural Sciences, Zhengzhou, Henan 450002, China

* Corresponding authors: Fang Wei, E-mail address: [fangwei@zzu.edu.cn](mailto:fangwei@zzu.edu.cn); and Baoming Tian, E-mail address: [tianbm@zzu.edu.cn](mailto:tianbm@zzu.edu.cn).

**Supplementary Figures:**


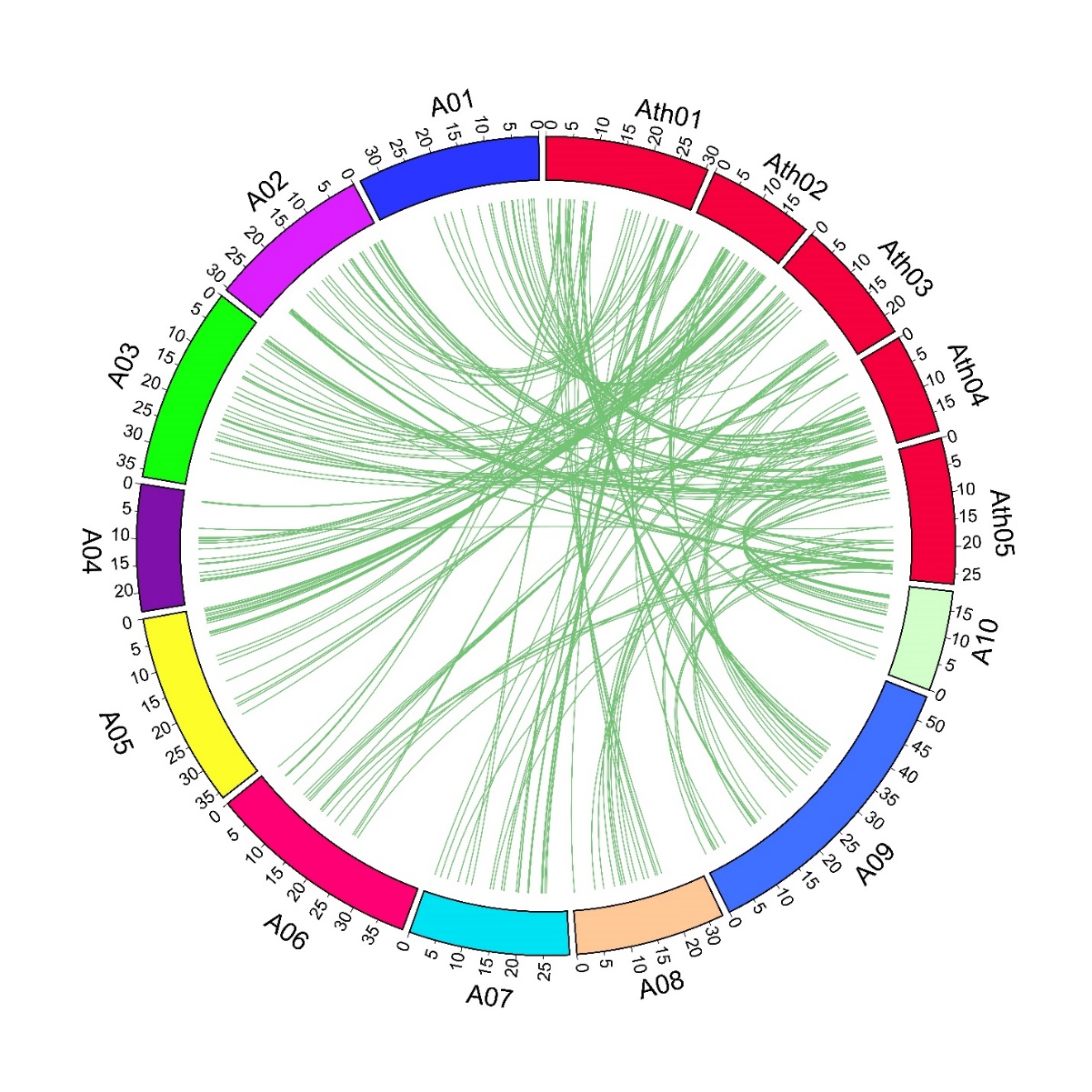


Figure S1. Gene duplication and collinearity analysis among Flower time genes from including B.rapa and A.thaliana. Lines connecting genes depict ortholog pairs diverged from the same ancestor. A1-A10 indicated *B.rapa* chromosomes, and Ath01-Ath05 displayed *A.thaliana* chromosomes.


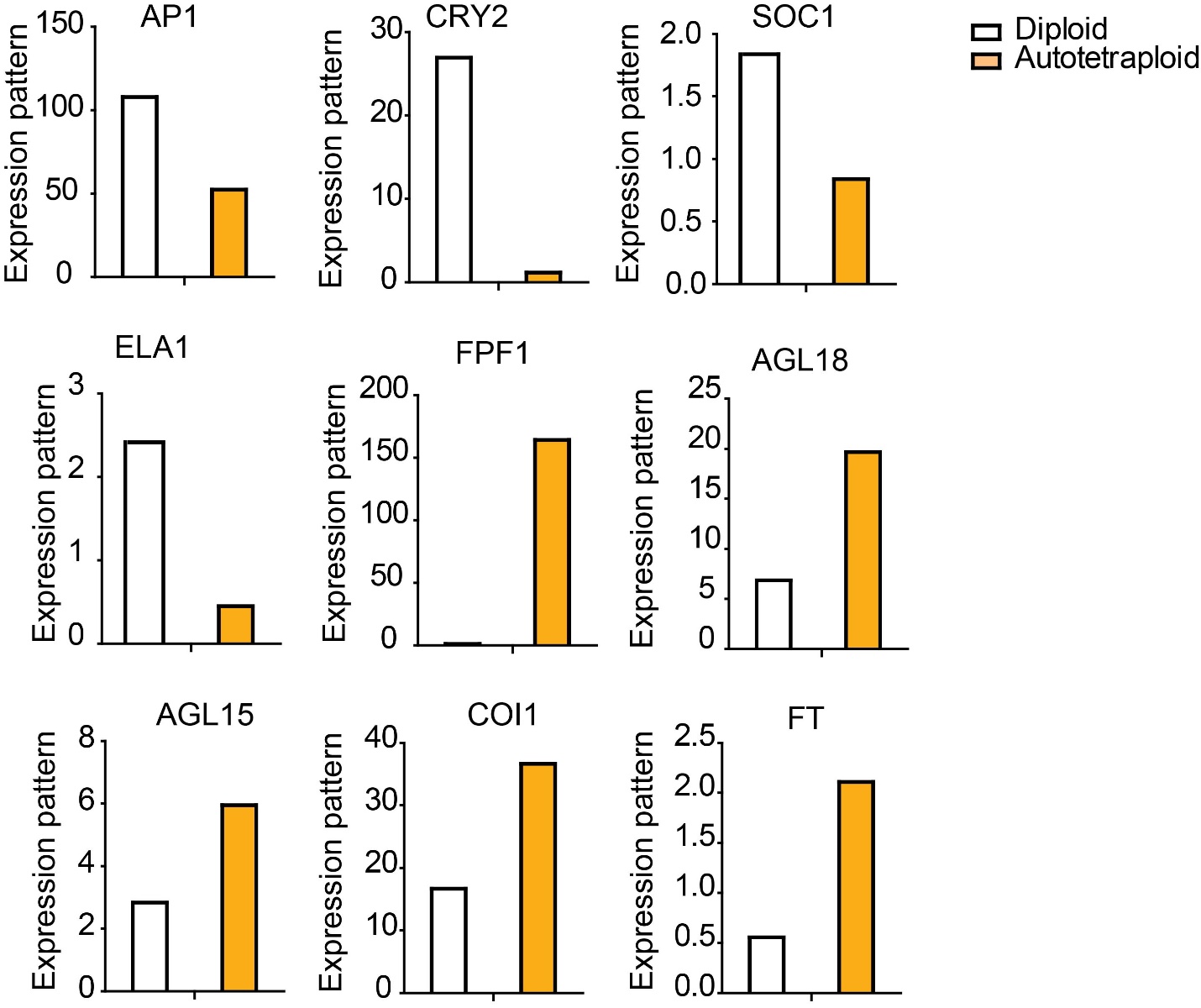


Figure S2. RNA-seq results of key genes in the flowering time pathway.
